# A Case of Pediatric Pancreatic Insulinoma Diagnosed 4 Years after the Onset

**DOI:** 10.70352/scrj.cr.25-0355

**Published:** 2025-10-28

**Authors:** Takazumi Kato, Souji Ibuka, Yuki Sengoku, Tatsuki Kawahara, Hideki Matsumoto, Michio Ozeki, Hidenori Ohnishi, Nobuhisa Matsuhashi

**Affiliations:** 1 Department of Digestive Surgery and Pediatric Surgery, Gifu University Graduate School of Medicine, Gifu, Gifu, Japan; 2 Department of Pediatrics, Gifu University Graduate School of Medicine, Gifu, Gifu, Japan

**Keywords:** insulinoma, neuroendocrine, pancreas, child, NEN, NET, NEC

## Abstract

**INTRODUCTION:**

Insulinomas are rare tumors resulting in hyperinsulinemic hypoglycemia. Insulinomas are usually seen in adults, and are very rare in the pediatric population. A total of 10% of insulinomas that occur are associated with multiple endocrine neoplasia type 1, and overall, 10% are malignant.

**CASE PRESENTATION:**

A 12-year-old boy suffered from an absence-like condition, 4 years before presentation. It was later discovered that this was due to hypoglycemia. His fasting blood glucose level was normal (93 mg/dL), but his insulin level was 60.8 µIU/mL, and his C-peptide level was 4.25 ng/mL, at the first visit. Abdominal CT and MRI showed a 6-cm nodular lesion in the anterior part of the pancreatic tail. Somatostatin receptor scintigraphy revealed radiotracer accumulation in the tumor. There was no evidence of lymphadenopathy or distant metastasis. He underwent laparoscopic spleen-preserving distal pancreatectomy. Pancreatic pathology revealed a grade 2 neuroendocrine tumor and malignant insulinoma. Postoperatively, the patient had no further hypoglycemia. At 9 months after the operation, he was under careful follow-up observation.

**CONCLUSIONS:**

We present a pediatric case of a malignant insulinoma that was preoperatively diagnosed as benign and subsequently treated by minimally invasive surgery. There is no clear treatment strategy for pediatric malignant insulinomas. We suggest that—even if malignant insulinoma has been treated by minimally invasive surgery—follow-up with surveillance imaging is acceptable if curative resection has been achieved; however, further surgical intervention may be warranted in selected cases.

## Abbreviations

MEN1
: multiple endocrine neoplasia type 1
NEC
: neuroendocrine carcinoma
NEN
: neuroendocrine neoplasm
NET
: neuroendocrine tumor
panNETs
: pancreatic neuroendocrine tumors
WHO
: World Health Organization

## INTRODUCTION

Pancreatic neuroendocrine tumors (panNETs) are a rare subset of neoplasms that arise from neuroendocrine cells derived from gastrointestinal stem cells, which originate from the endoderm. In children and adolescents, the annual incidence of panNET is extremely low, estimated at less than 0.1 per million individuals. Among pediatric panNETs, insulinomas (a type of panNET) predominate, representing 50%–60% of functional tumors and are characterized by hypoglycemic symptoms due to excessive insulin production.^1)^ On the contrary, in all age groups, insulinomas affect 1–4 per 1 million population and are benign in 90% of cases.^2)^ PanNETs constitute a clinically significant uncommon malignancy.^1)^ However, insulinomas are distinct from other types of panNETs in that the majority are considered benign.

We herein present the case of a pediatric patient with a malignant pancreatic insulinoma in which the clinical course was marked by difficult treatment decisions.

## CASE PRESENTATION

The patient was a 12-year-old boy who suffered from an absence-like condition 4 years prior to his presentation. The attending pediatrician had determined that the loss of consciousness was due to epilepsy and was therefore unable to detect hypoglycemia. However, he reported that the symptoms were alleviated by eating candy. He developed the same symptoms while traveling and when he went to the hospital it was discovered that the cause was hypoglycemia.

**Fig. 1:**
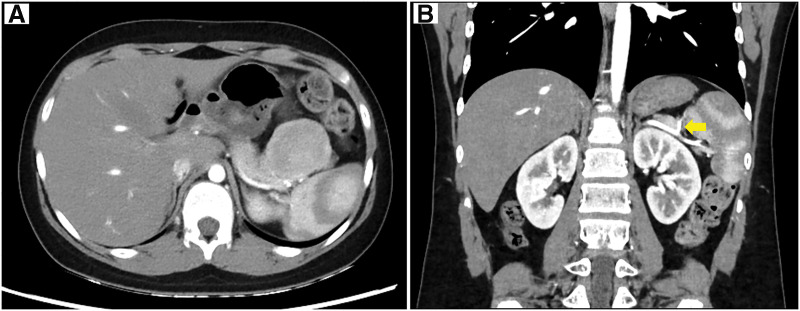
Abdominal CT. (**A**) Abdominal CT revealed a 6-cm nodular lesion in the anterior part of the pancreatic tail with strong contrast. (**B**) The tumor-feeding artery (arrow) branched off from the splenic artery.

**Fig. 2:**
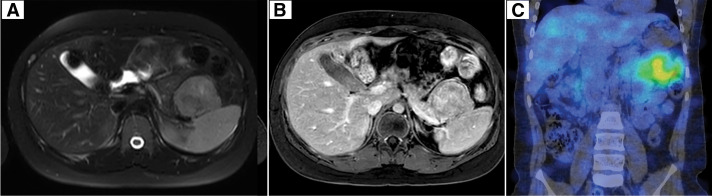
Abdominal MRI and somatostatin receptor scintigraphy. (**A**) Abdominal MRI revealed a T2-hyperintense 6-cm solid lesion in the anterior of the pancreatic tail. (**B**) Contrast-enhanced MRI revealed the T1-hypointense tumor with a rim of contrast enhancement. (**C**) Somatostatin receptor scintigraphy with ^111^In-pentetreotide (OctreoScan) showed homogeneous radiotracer uptake in the tumor.

At the first visit, his fasting blood glucose level was normal (93 mg/dL), but his insulin level was 60.8 μIU/mL, and his C-peptide level was 4.25 ng/mL. Abdominal CT showed a 6-cm nodular lesion in the anterior part of the pancreatic tail with strong contrast. No other masses were found in the pancreas, and it was considered to be a solitary tumor (**Fig. 1**). Abdominal MRI showed that the tumor was hypointense on T1-weighted imaging and hyperintense on T2-weighted imaging. Contrast-enhanced MRI showed enhancement of the tumor rim. Somatostatin receptor scintigraphy with ^111^In-pentetreotide (OctreoScan) revealed radiotracer accumulation in the tumor (**Fig. 2**). There was no evidence of lymphadenopathy or distant metastasis. We diagnosed the tumor as a benign insulinoma and planned to perform minimally invasive surgery.

**Fig. 3:**
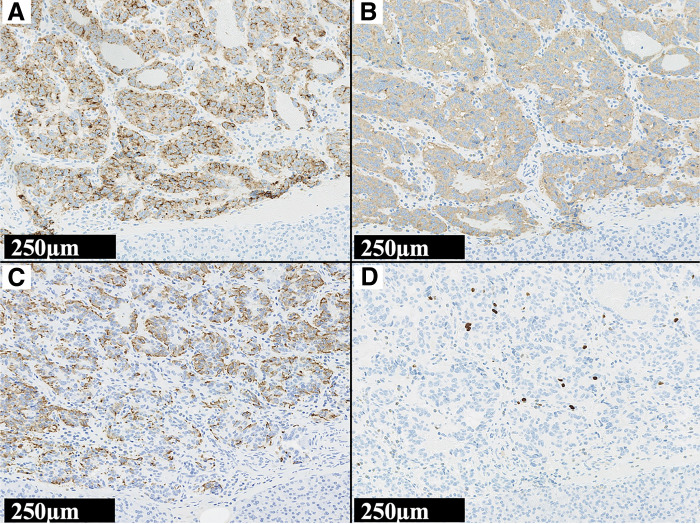
The tumor’s microscopic sections. Histopathology of the insulinoma. The resected tumor was positive for anti-chromogranin A antibody (**A**) and anti-synaptophysin antibody (**B**), and the tumor was a neuroendocrine tumor. The tumor was positive for anti-insulin antibody (**C**), that was insulinoma. Tumor grade was assessed using Ki67-index according to the World Health Organization guideline, and Ki67-index was 3.4% (**D**). The tumor was a grade 2 neuroendocrine tumor.

**Fig. 4:**
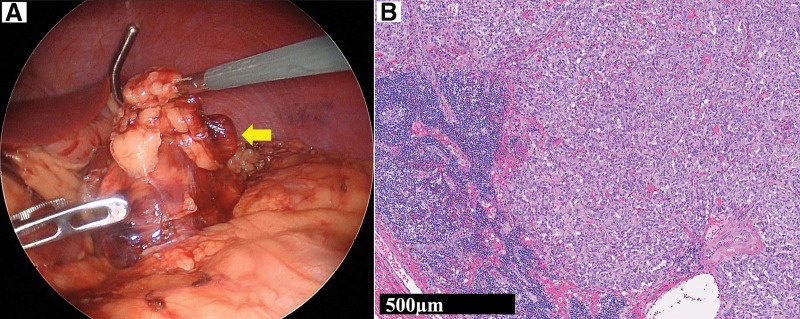
Regional lymph node metastasis. (**A**) During surgery, a swollen regional lymph node (arrow) was found in the fatty tissue near the tumor. (**B**) Histopathological examination revealed tumor cells in the lymph nodes.

He underwent a laparoscopic spleen-preserving distal pancreatectomy. Pancreatic pathology revealed a grade 2 neuroendocrine tumor (NET G2) with vascular and lymphovascular invasion, and a Ki-67 positivity rate of 3.4%. Staining of the pancreatic specimen with an anti-insulin antibody confirmed the presence of insulinoma (**Fig. 3**). Although preoperative imaging tests did not reveal any lymph node metastasis, a swollen regional lymph node was detected and removed during surgery. Histopathological examination revealed tumor cells in the lymph node (**Fig. 4**).

Postoperatively, the patient had no further hypoglycemia. With a final diagnosis of malignant insulinoma, genetic testing for multiple endocrine neoplasia type 1 (MEN1), as well as additional distal pancreatectomy and regional lymph node dissection were discussed with the patient and his parents, but were not pursued at the time. MRI at 5 months postoperatively revealed no evidence of local recurrence. At 9 months after the surgery, he remained free of hypoglycemia or loss of consciousness and was under careful follow-up observation.

## DISCUSSION

Hyperinsulinemic hypoglycemia in children is most commonly due to congenital hyperinsulinism resulting from defects in genes that regulate insulin secretion; there have been very few reports on pediatric acquired insulinomas.^2)^ Gupta et al. alidentified only 33 cases involving a child or adolescent with an insulinoma in a review of the relevant literature from the past 50 years.^3)^ For our patient, the sudden onset of symptoms without a history of neonatal hypoglycemia was consistent with an acquired process rather than a congenital one.

Insulinomas are rare tumors that result in hyperinsulinemic hypoglycemia. In all ages, insulinomas affect 1–4 per 1 million population and are benign in 90% of cases.^2)^ Almost all insulinomas occur within the pancreatic parenchyma. A total of 10% of insulinomas are associated with MEN1, and overall, 10% are malignant. Insulinoma is a classic example of a functional pancreatic neuroendocrine neoplasm (NEN). The 2022 World Health Organization (WHO) Classification of Endocrine and Neuroendocrine Tumors classifies epithelial NENs as well-differentiated (NET) or poorly differentiated (neuroendocrine carcinoma [NEC]).^4)^ Among NENs, which are rare malignant tumors, insulinomas have an important distinction in that many are benign. Based on the Ki-67 labeling index, the tumor was diagnosed as NET G2 (well-differentiated NEN, intermediate grade) according to the WHO classification.

Individuals with insulinomas commonly report adrenergic symptoms, including anxiety, tremulousness, palpitations, sweating, and hunger, as well as neuroglycopenic symptoms, such as confusion, seizures, and focal neurologic deficits.^3)^ The patient’s loss of consciousness caused by hypoglycemia was mistakenly thought to be due to epilepsy for 4 years prior to his diagnosis.

In patients with suspected insulinomas, imaging is required for the diagnosis, and multiple modalities may be necessary, including ultrasound, endoscopic ultrasound, MRI, CT, and PET.^2)^ However, owing to the small size of most insulinomas, they are not always detected by imaging. Even if no tumor is found, abdominal imaging will need to be repeated at intervals until a diagnosis can be made. The majority of endocrine pancreatic tumors can be visualized by somatostatin receptor scintigraphy with an OctreoScan. The OctreoScan is more likely to visualize primary tumors, as well as lymph node and bone metastases, but is less successful in detecting liver metastases.^5)^ If an insulinoma is visualized, the preferred treatment is tumor enucleation.^3)^

The differences in clinical presentations and outcomes between benign and malignant insulinomas are not well understood, and there is no consensus on the diagnostic criteria for malignant insulinomas. Malignancy was defined based on the presence of lymphovascular invasion, positive regional lymph nodes, direct invasion into surrounding peripancreatic tissue, or the presence of distant metastases. In our case, the only evidence that his insulinoma was malignant was the presence of positive lymph nodes. The optimal approach when malignant insulinoma is suspected preoperatively is to proceed with an oncological complete resection along with regional lymph node dissection.^6)^ The recommendation for the systematic removal of lymph nodes in the peritumoral area during any panNET operation is supported.^7)^ We preoperatively diagnosed the tumor as a benign insulinoma and selected minimally invasive surgery, but the pathological examination revealed malignant findings. However, the findings regarding additional surgeries are controversial. Sada et al. reported that “if patients were diagnosed with malignant insulinoma based on the final pathology report after enucleation or in cases in which lymph node dissection was not performed, the patient was followed with surveillance imaging rather than additional surgical treatment”.^6)^

The number and presence of positive lymph nodes have important prognostic value in patients with pancreatic neuroendocrine tumors, whereas lymph node metastases of intrapancreatic insulinomas are unlikely to affect the patient prognosis.^7)^

When managing pediatric insulinomas, we need to consider the involvement of MEN1. In addition to individuals with pancreatic tumors, those with MEN1 are also at risk for pituitary tumors, parathyroid hyperplasia, and dermatologic manifestations. Genetic testing is the gold standard for establishing the diagnosis of MEN1.^3)^ We explained to the patient and his parents that genetic testing for MEN1 would allow screening and early intervention for diseases that might develop in their family in the future; however, they did not wish to undergo additional surgery or genetic testing for MEN1. To monitor and manage recurrent insulinoma, we recommend patient education on hypoglycemic symptoms, monitoring for hypoglycemia and hyperinsulinemia, and annual imaging.

## CONCLUSIONS

We present a pediatric case of a malignant insulinoma that was preoperatively diagnosed as benign and subsequently treated by minimally invasive surgery. Pediatric malignant insulinomas are very rare tumors, and there is no clear treatment strategy. We suggest that—even if malignant insulinoma has been treated by minimally invasive surgery—follow-up with surveillance imaging is acceptable if curative resection has been achieved; however, further surgical intervention may be warranted in selected cases.

## DECLARATIONS

### Availability of data and materials

The availability of the data used in this case is subject to confirmation by the authors. For more information on data availability and access procedures, please contact the corresponding author.
